# Supplementary material for: Investigations on the Morphological, Mechanical, Ablative, Physical, Thermal, and Electrical Properties of EPDM-Based Composites for the Exploration of Enhanced Thermal Insulation Potential
Source: Polymers (Basel). 2022 Feb 22;14(5):863. doi: 10.3390/polym14050863 (PMC8912367; doi:10.3390/polym14050863)
Supplement: Supplementary file 1 [file polymers-14-00863-s001.zip › polymers-1593844-supplementary-done.pdf]

polymers

MDPI

Supplementary Material

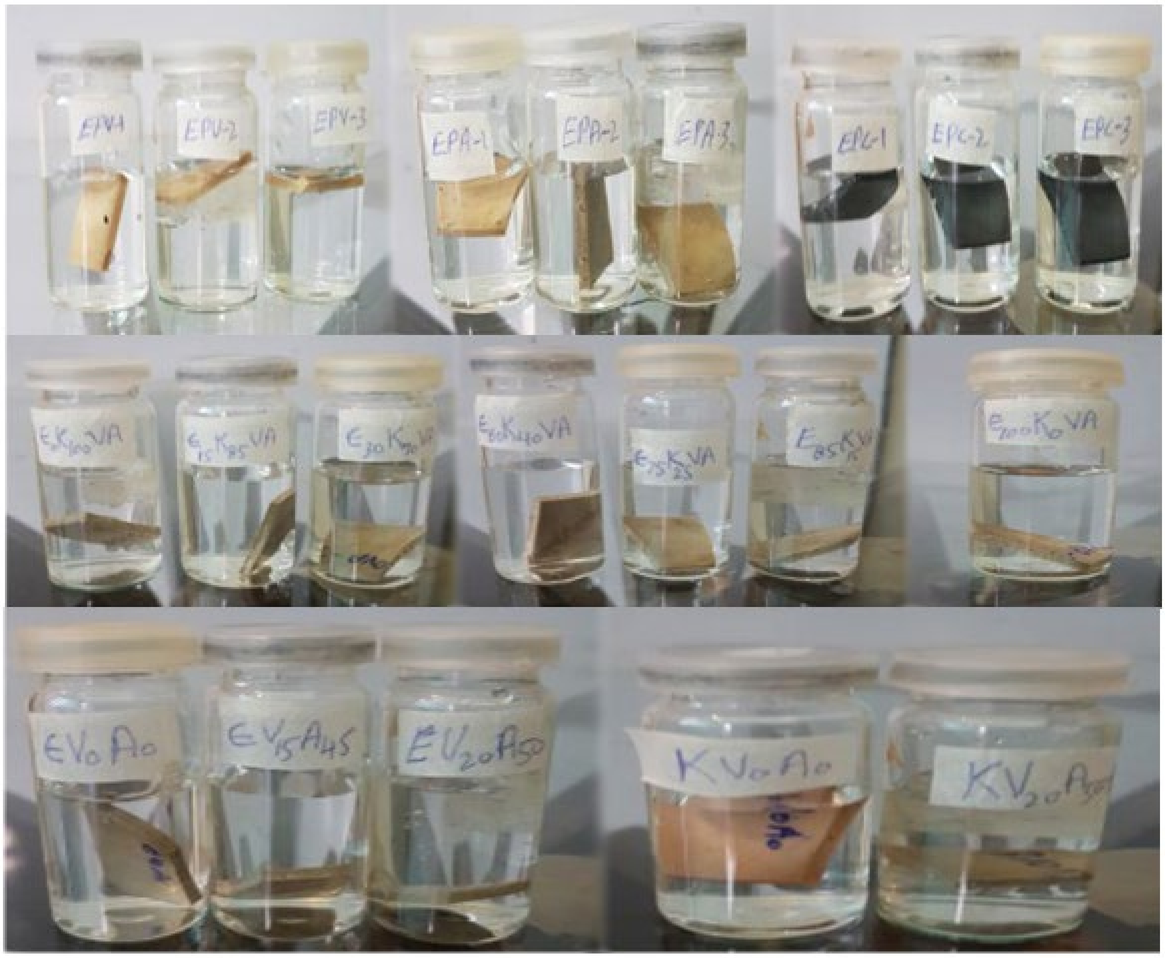

Figure S1: Pictures of experimental setup for swelling index measurement of phase-1 (top), phase-2 (mid), and phase-3 (bottom) samples.

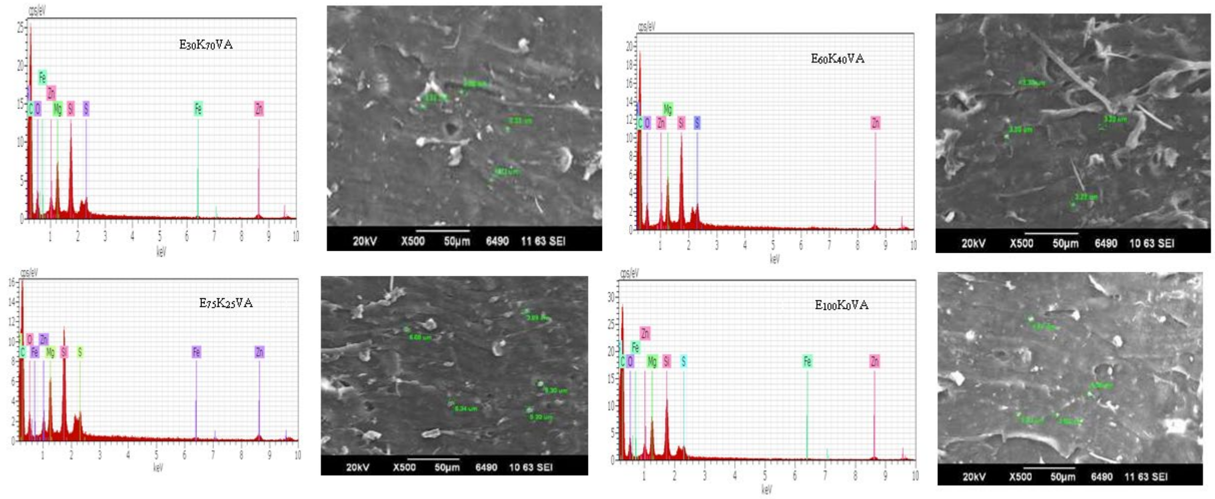

Figure S2: EDS spectra and SEM images of some samples of phase-2 composites.

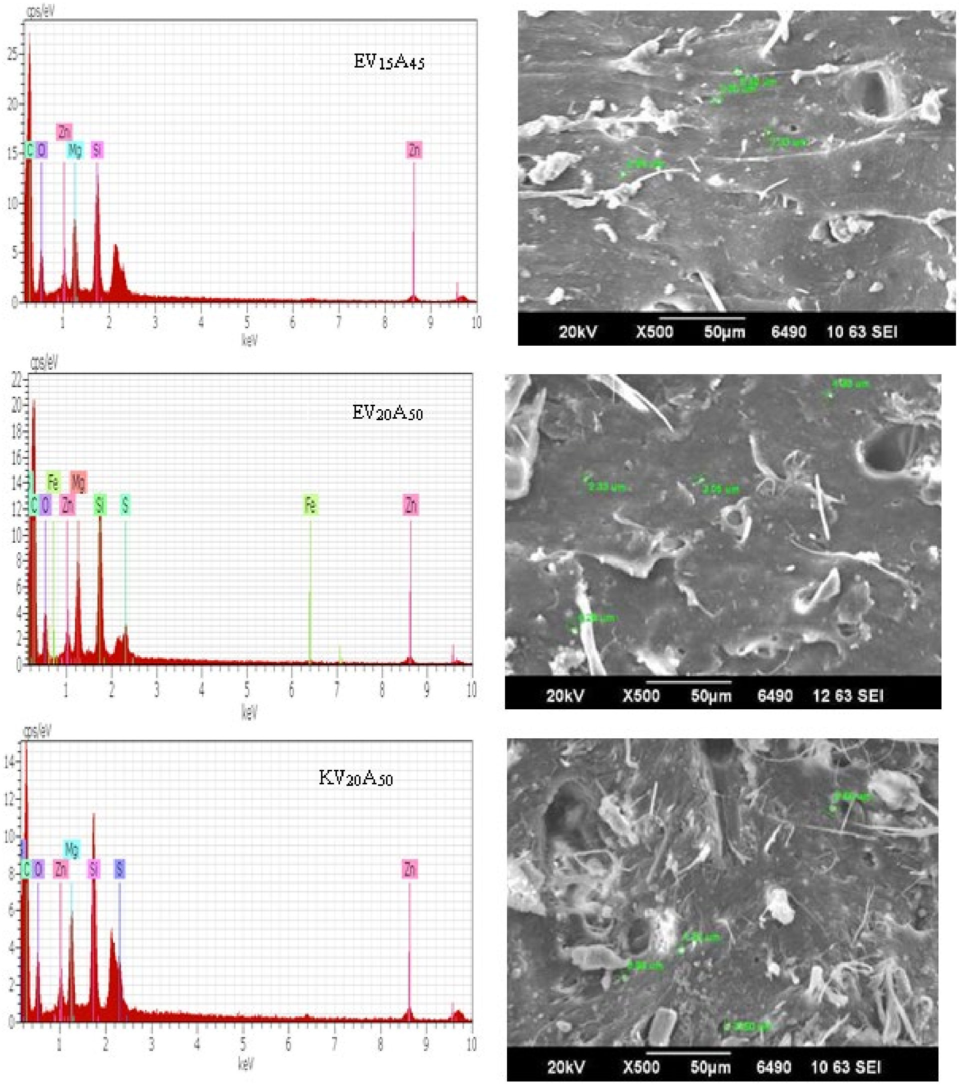

Figure S3: EDS spectra and SEM images of some samples of phase-3 composites.

Table S1: Tensile stress and tensile strain values of phase -1, -2 and -3 composites.

Phase-1 samples	EPV-1	EPV-2	EPV-3	EPA-1	EPA-2	EPA-3	EPC-1	EPC-2	EPC-3
Stress (MPa)	1.87	2.20	5.60	1.28	1.72	2.73	0.90	1.23	1.44
Strain (%)	445	454	855	208	169	128	219	203	262
Phase-2 samples	E_0K_{100} VA	$E_{15}K_{85}$ VA	$E_{30}K_{70}$ VA	$E_{60}K_{40}$ VA	$E_{75}K_{25}$ VA	$E_{85}K_{15}$ VA	$E_{100}K_0$ VA		
Stress (MPa)	2.63	2.56	3.01	3.42	3.08	3.25	3.89		
Strain (%)	338	314	242	138	654	169	774		
Phase-3 samples	EV_0A_0	$EV_{20}A_{50}$	$EV_{15}A_{45}$	KV_0A_0	$KV_{20}A_{50}$				
Stress (MPa)	1.13	4.07	3.16	0.50	3.49				
Strain (%)	161	294	654	104	164				

Table S2: Linear and mass ablation values of phase-1, -2 and -3 composites.

Phase-1 samples	EPV-1	EPV-2	EPV-3	EPA-1	EPA-2	EPA-3	EPC-1	EPC-2	EPC-3
Linear ablation (mm/s)	0.42	0.46	0.35	0.06	0.07	0.11	0.13	0.11	0.12
Mass ablation (g/s)	0.15	0.17	0.13	0.04	0.04	0.04	0.08	0.06	0.06
Phase-2 samples	E_0K_{100} VA	$E_{15}K_{85}$ VA	$E_{30}K_{70}$ VA	$E_{60}K_{40}$ VA	$E_{75}K_{25}$ VA	$E_{85}K_{15}$ VA	$E_{100}K_0$ VA		
Linear ablation (mm/s)	0.08	0.10	0.12	0.14	0.16	0.12	0.10		
Mass ablation (g/s)	0.04	0.04	0.05	0.05	0.05	0.05	0.05		
Phase-3 samples	EV_0A_0	EV_{20} A_{50}	EV_{15} A_{45}	KV_0 A_0	KV_{20} A_{50}				
Linear ablation (mm/s)	0.41	0.12	0.11	0.50	0.12				
Mass ablation (g/s)	0.15	0.05	0.05	0.17	0.05				

Table S3: Swelling index values of phase -1, -2 and -3 composites.

Phase-1 samples	EPV-1	EPV-2	EPV-3	EPA-1	EPA-2	EPA-3	EPC-1	EPC-2	EPC-3
S.I (%)	162	161	145	188	176	150	230	208	185
Phase-2 samples	E_0K_{100} VA	$E_{15}K_{85}$ VA	$E_{30}K_{70}$ VA	$E_{60}K_{40}$ VA	$E_{75}K_{25}$ VA	$E_{85}K_{15}$ VA	$E_{100}K_0$ VA		
S.I (%)	79	98	106	129	167	170	174		
Phase-3 samples	EV_0A_0	EV_{20} A_{50}	EV_{15} A_{45}	KV_0 A_0	KV_{20} A_{50}				
S.I (%)	217	183	142	181	63				

Table S4: Amount of material left (%) in TGA analysis of phase -1, 2 and 3 composites at 500°C.

Phase-1 samples	EPV-1	EPV-2	EPV-3	EPA-1	EPA-2	EPA-3	EPC-1	EPC-2	EPC-3
Material Left (%)	14.6	18.0	26.4	17.3	15.7	29.6	15.8	23.4	29.3
Phase-2 samples	E_0K_{100} VA	$E_{15}K_{85}$ VA	$E_{30}K_{70}$ VA	$E_{60}K_{40}$ VA	$E_{75}K_{25}$ VA	$E_{85}K_{15}$ VA	$E_{100}K_0$ VA		
Material Left (%)	61.2	65.4	69.4	76.0	81.3	83.7	91.0		
Phase-3 samples	EV_0A_0	EV_{20} A_{50}	EV_{15} A_{45}	KV_0 A_0	KV_{20} A_{50}				
Material Left (%)	7.4	32.4	29.8	6.2	31.9				
